# Exploring the phytoconstituents, antimicrobial potency, and cytotoxic effects of essential oil from *Origanum punonense* from Palestine

**DOI:** 10.1186/s12906-024-04395-4

**Published:** 2024-02-28

**Authors:** Kamal Issa, Amjad Bakhatan, Majde Abu Khaled, Nidal Jaradat, Mohammed Hawash, Nawaf Al-Maharik, Mustafa Ghanim, Mohammad Qadi

**Affiliations:** 1 Department of Medicine, Faculty of Medicine and Health Sciences, An-Najah National University, P.O. Box 7, Nablus, Palestine; 2 Department of Pharmacy, Faculty of Medicine and Health Sciences, An-Najah National University, P.O. Box 7, Nablus, Palestine; 3 Department of Chemistry, Faculty of Sciences, An-Najah National University, P.O. Box 7, Nablus, Palestine; 4 Department of Biomedical Sciences, Faculty of Medicine and Health Sciences, An-Najah National University, P.O. Box 7, Nablus, Palestine

**Keywords:** *Origanum punonense*, Essential oil, Antimocrobial, Diabetic foot, Antiproliferative

## Abstract

**Background:**

*Origanum punonense* Danin is one of the old traditional medicinal plants Bedouins utilize in the Dead Sea region to treat a variety of illnesses, those caused by infections. The current study aimed to identify *the phytochemical components of O. punonense* essential oil (EO) and determine its antiproliferative and antimicrobial effects.

**Methods:**

Gas chromatography and mass spectrometry were employed to detect the phytochemical constituents of *O. punonense* EO. Broth microdilution assay was utilized to determine the antimicrobial effects against various microbial species, including those causing diabetic foot infections.

**Results:**

This study revealed that *O. punonense* EO contains 44 phytochemical compounds, of which 41 compounds were detectable and amounted to 99.78% of the total oil. The main chemical components of the oil were carvacrol (57.4%), p-cymene (6.66%), carvone (5.35%), pinene (4.9%), and terpinene (2.96%). The antiproliferative activity of different concentrations of *O. punonense* EO was noted in all of the investigated cell lines, with the best activity at the concentration of 500 µg/mL. The greatest antibacterial activity was against *Staphylococcus aureus*, *Escherichia coli*, *Klebsiella pneumoniae*, and *Proteus vulgaris*, with MIC values of 1.56 µL/mL. In addition, and the *O. punonense* EO showed strong antifungal activity against *Candida albicans* with a MIC value of 0.8 µL/mL. In addition, the *O. punonense* EO showed potent antibacterial activity against all MRSA samples obtained from the diabetic foot with a MIC value of 3.13 µL/mL. The *O. punonense* EO demonstrated potent activity against Carbapenem-resistant *Enterobacterales*, *Citrobacter freundii*, and *K. pneumoniae*, with MICs value of 6.25 µL/mL.

**Conclusion:**

The potent antiproliferative and broad antimicrobial activity of *O. punonense* EO makes it an effective strategy for treating infections, especially in immunocompromised patients with chronic comorbidities such as cancer and diabetes mellitus.

## Introduction

Herbal medicine is a traditional medicine used in every culture worldwide [1]. In some countries, herbal medicines may contain, by tradition, natural organic or inorganic active ingredients that are not of plant origin (e.g., animal and mineral materials). Herbs include crude plant material, such as leaves, flowers, fruit, seeds, stems, wood, bark, roots, rhizomes, or other plant parts, which may be entire, fragmented, or powdered. Herbal materials include, in addition to herbs, fresh juices, gums, fixed oils, essential oils, resins, and dry powders of herbs [2]. Essential oils (EOs) are extracted from different parts of plants, and most of them are liquid mixtures of volatile components, including monoterpenes, sesquiterpenes, and others [3]. EOs play an important role in medicine due to their antibacterial, antiviral, antifungal, anti-inflammatory, anticancer, and anthelmintic activities [4, 5].

Labiatae (Lamiaceae ) is a family of flowering plants that includes 236 genera and more than 7000 species and is distributed nearly worldwide [6]. Most of this family are perennial or annual herbs with square stems, and some species may have woody shrubs or subshrubs that are fragrant and rich in EOs [7].

The morphological description of *Origanum* is medium-size, subshrub by Labiatae, with subsessile, ovate, glandular punctate leaves and paniculate inflorescences, few-flowered verticillasters arranged in dense spikes with distinct, often colored, bracts, calyces variable: 5-toothed, subregular, 2-lippe, or 1-lipped with developed or reduced teeth, corollas 2-lipped, sometimes saccate or flattened [8].

*Origanum punonense* Danin is native to Palestine and grows in grassy and rocky habitats. Due to its pleasant aromatic odor and taste, Bedouins use it in the Dead Sea region as a culinary herb and an ornamental plant. It is utilized in traditional Arabic medicine to cure infectious skin diseases, respiratory infections through vapor inhalation, and several GIT conditions. *O. punonense* has white and labiateous small flowers with a yellow center. It has black-colored seeds with a round and small shape [9, 10].

Microbial infections, cancer, and diabetic foot are distinct medical illnesses with independent pathophysiological pathways, but they do have some parallels in their effects on human health [11, 12]. Microbial infections occur when harmful germs, mainly bacteria, invade and multiply within the host’s tissues [13]. On the other hand, cancer is distinguished by the unrestrained multiplication of cells and the possibility of developing into a harmful state, resulting in the creation of abnormal growths known as tumors [14]. Diabetic foot is a complication of diabetes mellitus that results from a combination of variables such as decreased wound healing, neuropathy, and reduced blood flow. This leads to chronic ulcers and increases the risk of infections [15]. Although originating from different sources, all three illnesses can provoke inflammatory reactions, leading to tissue damage and systemic repercussions [16]. Gaining insight into the fundamental mechanisms of these diseases is essential for developing specific therapeutic approaches and prevention strategies. Furthermore, multidisciplinary research endeavors are crucial for investigating novel medical agents to treat microbial infections, cancer, and diabetic foot, facilitating the formulation of comprehensive strategies to tackle their clinical complexities.

Therefore, the current study aims to characterize the chemical compositions and evaluate the antimicrobial activity against a series of harmful strains, with an emphasis on those causing diabetic foot complications and the cytotoxic effects of *O. punonense* EO collected from the shores of the Dead Sea in the Jericho region of Palestine.

## Materials and methods

### Plant material and essential oil extraction

The leaves of *O. punonense* were gathered in February 2023 from the shores of the Dead Sea in the Jericho region of Palestine. The herb was identified by Pharmacologist Dr. Nidal Jaradat in the Pharmacognosy Laboratory/An-Najah National University under the code number (Pharm-PCT-1731). The leaves were separated carefully, washed twice with distilled water, dried for about 15 days in the shade at room temperature, grounded well, and stored in cloth bags for future use.

The *O. punonense* EO was extracted using the Microwave-ultrasonic method described by Jaradat et al. [17]. However, in the extraction process, the powder suspension was exposed to ultrasonic waves to improve the EO extraction process. A 1 L round-bottom flask containing about 100 g of the dried powdered leaves was placed in this apparatus. The powder was suspended in about 500 ml of deionized water in this flask. Then, the flask was connected to the Clevenger apparatus, which was placed in the same apparatus. While carrying out the extraction process, the microwave-ultrasonic extractor apparatus power was adjusted at 1000 W. The ultrasonic power of the apparatus was adjusted at its maximum power as well (40 kHz). This apparatus’s extraction process was conducted for 30 min at 100 ˚C. This procedure was repeated three times for the plant sample. The obtained *O. punonense* EO was collected into a clean beaker, chemically dried using magnesium sulfate, and stored in the refrigerator at 2–8˚C until use. The average yield of the EO was 0.71% (w/w) [17].

### Gas chromatography/mass spectrometry (GC-MS)

GC-MS chromatograms were recorded using the Shimadzu QP-5000 apparatus (Columbia, Maryland, U.S.A.). GC-MS was equipped with an Rtx-5ms column (0.25 mm thickness, 30 m long, and 0.250 mm internal diameter). Helium gas was used as a carrier at a standard flow rate of 1 ml//min. The injector temperature was programmed at 220 °C while the oven temperature was adjusted from 50 °C (1 min hold) at 5 °C/min to 130 °C, then at 10 °C/min to 250 °C and kept isothermally for 15 min. The transfer line temperature was set to 290 °C. An electron ionization system was used for GC-MS detection with detector volts of 1.7 KV. A scan rate of 0.5s and a scan speed of 1000 amu/sec were applied, covering a mass range from 38 to 450 M/Z. The reference spectra in the mass spectrometry data center of the National Institute of Standards and Technology (NIST) were used to identify the chemical components of the EO, also by comparing their retention indices and Kovats index in the literature. The quantitative data were electronically obtained from the integrated peak [18].

### Antimicrobial evaluation

#### Microorganisms

##### ATCC strains

The antibacterial effect of the *O. punonense* EO was determined using five bacterial strains, obtained from the American Type Culture Collection (ATCC), including *Pseudomonas aeruginosa* (ATCC 9027), *Escherichia coli* (ATCC 25,922), *Klebsiella pneumoniae* (ATCC 13,883), *P. vulgaris* (ATCC 8427) and *Staphylococcus aureus* (ATCC 25,923). The antifungal activity of the *O. punonense* EO was evaluated against the growth of *Candida albicans* (ATCC 90,028).

##### Clinical bacterial strains

Eight bacterial strains that were clinically isolated and identified from patients with diabetic foot disease were employed in this investigation to assess the antibacterial activity of the *O. punonense* EO. Three isolates were gram-negative bacteria, specifically *Klebsiella pneumoniae*, *Pseudomonas aeruginosa*, and *Citrobacter freundii*, while the remaining five were methicillin-resistant *Staphylococcus aureus* (MRSA). All the clinical strains utilized in our study were isolated and identified at the Laboratory Department of Najah National University Hospital, where they employ the VITEK 2 system (bioMérieux) for microbe identification and antimicrobial sensitivity testing.

### Antimicrobial assays

The broth microdilution method described elsewhere was used in this study to evaluate the antimicrobial activity of the EO of *O. punonense* bacteria strains cultured for 18–24 h in a culture plate. The EO was dissolved in 5% DMSO at a 200 µL/mL concentration. After being filter-sterilized, the produced *O. punonense* EO solution was serially micro-diluted (by two folds) 10 times in sterile Muller Hinton broth (MHB) (RPMI media for *C. albicans.* The dilution procedures were carried out on 96-well plates under aseptic conditions. In the 96-well plate, micro-well number 11 contained *O. punonense* EO-free MHB, used as a positive control for bacterial growth. On the other hand, micro-well number 12 included *O. punonense* EO-free MHB that was left un-inoculated with any of the tested bacterial cells. This well was used as a negative control for microbial growth. Micro-wells numbers 1 to 11 were inoculated aseptically with the test microbes prepared using fresh microbial inoculum suspended in MHB. At the time of inoculation, the ultimate microbial cell concentrations were around 5 × 10^5^ colony-forming units (CFU)/mL for the investigated bacterial isolates. The antimicrobial activity of the *O. punonense* against each of the tested organisms was evaluated in triplicate. All inoculated plates were incubated at 35 °C. The incubation period lasted about 24 h (48 h for *C. albicans*, and the minimum inhibitory concentration (MIC) of the tested *O. punonense* volatile oil was the lowest concentration at which no discernible microbial growth in that micro-well was seen. Ciprofloxacin was used as a reference antibacterial activity control for our method, while fluconazole was used as a reference antifungal activity control [19, 20].

### Cell culture and cytotoxicity assay

The breast cancer (MCF-1), liver (Hep-G2, Hep-3B), cervical (HeLa), colon (Caco-2), and (hepatic stellate) LX-2 cells lines were cultured separately in RPMI medium and supplemented with 1% L-glutamine, 1% Penicillin/Streptomycin, and 10% fetal bovine serum. Cells were grown at 37 °C in a humidified atmosphere with 5% CO_2_ and were divided at 2.6 × 10^4^ cells/well in a 96-well plate. After 24 h, cells were incubated with various concentrations of the tested *O. punonense* EO. Cell viability was assessed by the CellTiter 96® Aqueous One Solution Cell Proliferation (MTS) Assay according to the manufacturer’s instructions (Promega Corporation, Madison, WI). Briefly, at the end of the treatment, 20 µl of MTS solution per 100 µl of media was added to each well and incubated at 37 °C for 2 h. Absorbance was measured at 490 nm [5].

### Statistical analysis

The mean values ± SD of standard deviations of all the findings of the *O. punonense* EO (antimicrobial and cytotoxic activities) were calculated, and the results with a p-value of < 0.05 were deemed significant. The cytotoxic experiments were done in duplicate. The unpaired *t*-test was used to analyze the data.

## Results

### Phytochemical characterizations

**Fig. 1:**
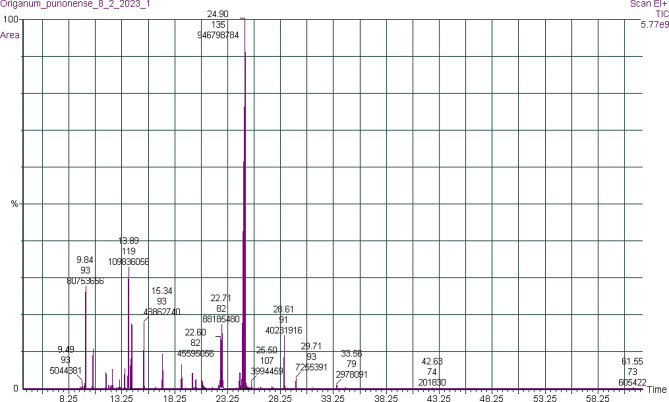
Gas chromatography-mass spectrometry chromatograph of *O. punonense* essential oil

The GC-MS identification technique results revealed that *O. punonense* EO contains 44 molecules, from which 41 components were detectable and identified, amounting to 99.78% of the total oil, as shown in Fig. 1. The main chemical components of the oil are carvacrol (57.4%), p-cymene (6.66%), carvone (5.35%), α-pinene (4.9%), and terpinene (2.96%). Forty-one components were classified according to their phyto-organic group. Oxygenated monoterpenes (74.95%) and monoterpene hydrocarbons (21.37%) were the major classes, as shown in Table 1.

**Table 1:** Phytochemical components of the EO (%), retention time (RT), and retention index (RI) extracted from *O. punonense* characterized by GC-MS apparatus

Compounds	RT	RI	Area	% Area
Tricyclene	9.31	920	720,260	0.04
α-Thujene	9.49	924	5,044,381	0.31
ND	9.72	929	3,025,890	0.18
α-Pinene	9.84	932	80,753,656	4.90
α-Fenchene	10.52	947	29,269,830	1.77
Camphene	10.7	951	632,678	0.04
β-Pinene	11.76	974	11,230,685	0.68
Oct-1-en-3-ol	12	980	2,462,992	0.15
3-octanone	12.17	983	1,188,587	0.07
Myrcene	12.36	988	13,079,083	0.79
Octan-3-ol	12.77	997	568,156	0.03
m-Mentha-1(7),8-diene	12.94	1000	498,601	0.03
δ-2-Carene	13.04	1003	6,230,455	0.38
δ-3-Carene	13.16	1006	240,792	0.01
α-Terpinene	13.52	1014	14,512,632	0.88
p-Cymene	13.89	1023	109,886,056	6.66
Limonene	14.08	1027	30,302,694	1.84
1,8-Cineole	14.19	1030	46,887,008	2.84
δ-Terpinene	15.34	1057	48,862,740	2.96
Terpinolene	16.45	1083	1,052,534	0.06
ρ-Cymenene	16.62	1087	198,446	0.01
Linalool	17.1	1098	26,705,358	1.62
Camphor	18.89	1144	22,466,356	1.36
Menthone	19.26	1154	409,562	0.02
Terpinen-4-ol	20.24	1179	7,776,528	0.47
Dihydro carveol	20.83	1194	17,909,766	1.09
trans-Dihydro carvone	21.12	1201	1,242,625	0.08
(Z)-Ocimenone	21.79	1220	788,686	0.05
Thymol, methyl ether	22.41	1237	2,390,820	0.14
trans-ocimenone	22.6	1243	45,595,056	2.76
Carvone	22.71	1246	88,185,480	5.35
Thymol	24.4	1293	24,081,318	1.46
Carvacrol	24.9	1307	946,798,784	57.40
Isodihydrocarveol acetate	25.5	1325	3,994,459	0.24
Eugenol	26.38	1352	1,002,688	0.06
α-Ylangene	27.16	1375	217,940	0.01
β-Bourbonene	27.43	1383	1,357,813	0.08
b-Elemene	27.6	1388	386,477	0.02
Methyl eugenol	27.91	1397	74,425	0.00
ND	28.1	1403	144,044	0.01
β-caryophyllene	28.61	1420	40,231,916	2.44
α-caryopyllene	29.71	1455	7,255,391	0.44
(E,E)-α-Farnesene	31.3	1506	458,884	0.03
Caryophyllene oxide	33.56	1583	2,978,091	0.18
ND	34.37	1610	315,107	0.02
**SUM**			164,941,570	100.00
**Yields**				0.71
**Phytochemical groups**				
Monoterpene hydrocarbons				21.37
Oxygenated monoterpenes				74.95
Sesquiterpene hydrocarbons				3.03
Oxygenated sesquiterpene				0.18
Others				0.26
ND				0.21
Total				100.00

ND: Not detected

### Antimicrobial activity

Table 2 shows the outcomes of O. *punonense* EO antibacterial activity against Gram-positive and Gram-negative bacteria and fungi strains. According to the findings, *O. punonense* EO exhibited potent antibacterial and antifungal properties against the examined fungal, Gram-positive, and Gram-negative strains. The greatest antibacterial activity was shown against *S. aureus*, *E. coli*, *K. pneumoniae*, and *P. vulgaris*, with MIC values of 1.56 µL/mL. In addition, against *P. aeruginosa* (ATCC), the antibacterial activity of the *O. punonense* EO showed MIC values of 3.13 µL/mL, and the *O. punonense* EO has strong activity against *C. albicans* with a MIC value of 0.8 µL/mL. Moreover, in the samples obtained from diabetic foot patients, the *O. punonense* EO showed a powerful effect against MRSA with a MIC value of 3.13 µL/mL for each sample of MRSA samples. The *O. punonense* EO demonstrated impressive activity against Carbapenem-resistant *Enterobacterales*, *C. freundii*, and *K. pneumoniae*, with MICs value of 6.25 µL/mL and weak effect on *P. aeruginosa* (clinical strain) with MIC value of 50 µL/mL.

**Table 2:** MIC values (µL/mL) of *O. punonense* EO, Ciprofloxacin and Fluconazole

Microbial strains						
		Source/ Number	Name	EO µL/mL	Ciprofloxacin µg/mL	Fluconazole µg/mL
Bacteria	Gram-positive	ATCC 25923	*S. aureus*	1.56	0.78	-
		Clinical strain	MRSA-1	3.13	15.6	-
		Clinical strain	MRSA-2	3.13	7.8	-
		Clinical strain	MRSA-3	3.13	15.6	-
		Clinical strain	MRSA-4	3.13	31.25	-
		Clinical strain	MRSA-5	3.13	15.6	-
	Gram-negative	ATCC 25922	*E. coli*	1.56	1.56	-
		ATCC 13883	*K. pneumoniae*	1.56	0.13	-
		ATCC 8427	*P. vulgaris*	1.56	15	-
		ATCC 9027	*P. aeruginosa*	3.13	3.12	-
		Clinical strain (*CRE)	*K. pneumoniae*	6.25	125	-
		Clinical strain (*CRE)	*C. freundii*	6.25	31.25	-
		Clinical strain	*P. aeruginosa*	50	62.5	-
Fungus		ATCC 90028	*C. albicans*	0.8	-	1.56

*CRE: Carbapenem-resistant *Enterobacterales*

### Antiproliferative activity

Table 3 shows the antiproliferative effect of *O. punonense* EO on CaCo-2, Hep3B, HeLa, MCF-7, HepG2, and LX-2 cell lines. The most effective antiproliferative activity was noted at the concentration of 500 µg/mL, as the EO had, roughly, a complete antiproliferative effect on CaCo-2, Hep3B, HeLa, MCF-7, HepG2, LX-2 cell lines with results of 95.9, 85.51, 90.1, 95.7, 91.05 and 97.6%, respectively.

**Table 3:** Antiproliferative effect of *O. punonense* EO on CaCo-2, Hep3B, HeLa, MCF-7, HepG2, and LX-2 cell lines

Conc. (µg/mL)	CaCo-2	Hep3B	HeLa	MCF-7	HepG2	LX-2
500	95.91 ± 0.28	85.51 ± 3.27	90.11 ± 0.77	95.74 ± 0.32	91.05 ± 3.18	97.66 ± 0.01
300	11.08 ± 0.83	21.16 ± 2.78	34.98 ± 1.13	19.51 ± 0.11	34.61 ± 1.41	24.18 ± 1.43
100	4.25 ± 0.64	8.08 ± 2.66	28.18 ± 0.09	0.00	33.14 ± 1.90	1.37 ± 0.83
50	9.52 ± 1.25	0.00	3.80 ± 0.50	0.00	29.97 ± 1.94	0.00
0	0.00	0.00	0.00	0.00	0.00	0.00

IC_50_ values indicate that the concentration of *O. punonense* EO is required to inhibit a biological process or response by 50% Caco-2, Hep3B, HeLa, MCF-7, HepG2, and LX2. Values are expressed in µg/ml, providing a standardized unit for the concentrations used in the experiments.

In Table 4, the IC_50_ values reveal a fascinating narrative of potency and inhibition of *O.* punonense on different cell lines used in the experiment. The *O. punonense* EO influence on Caco-2 cells manifested at an IC_50_ of 432.05 ± 3.65 µg/ml, where the delicate balance of cellular processes saw a significant impact. Hep3B cells exhibited a similar susceptibility, with an IC50 of 410.207 ± 4.79 µg/ml, marking the herbal influence on this cellular realm. In the realm of HeLa cells, the IC_50_ unveiled a nuanced interaction, standing at 323.556 ± 0.62 µg/ml, showcasing the distinctive sensitivity of this cell line to *O. punonense*. MCF-7 cells, known for their unique characteristics, responded at an IC_50_ of 379.997 ± 0.54 µg/ml, contributing another layer to the herb’s multifaceted pharmacological profile. HepG2 cells, a bastion of hepatic influence, displayed notable responsiveness with an IC_50_ of 235.913 ± 2.11 µg/ml, highlighting the differential impact of the herb on hepatocellular dynamics. Lastly, the LX-2 cells, representing a hepatic stellate cell line, revealed a significant IC_50_ of 389.856 ± 0.73 µg/ml, further enriching the tale of *O. punonense* cellular interactions. These IC_50_ values, expressed in micrograms per milliliter, provide quantitative insights into the herb’s efficacy and beckon further exploration into the nuanced effects across diverse cellular landscapes. All these findings were compared with the IC_50_ values of the positive control, 5-fluorouracil, against Caco-2, Hep3B, HeLa, MCF-7, HepG2, and LX2 cell lines, which were reported as 3.71 ± 0.84, 1.21 ± 1.0, 2.09 ± 1.01, 1.28 ± 0.45, 4.23 ± 1.78, and 0.73 ± 0.33 µg/mL, respectively, based on our recently published research [21].

**Table 4:** The *O. punonense* EO cytotoxicity IC_50_ values on Caco-2, Hep3B, HeLa, MCF-7, HepG2, and LX2 cells lines

Tests	IC_50_ (µg/mL)
	*O. punonense* EO
CaCo-2	432.05 ± 3.65
Hep3B	410.207 ± 4.79
HeLa	323.556 ± 0.62
MCF-7	379.997 ± 0.54
HepG2	235.913 ± 2.11
LX-2	389.856 ± 0.73

**Fig. 2:**
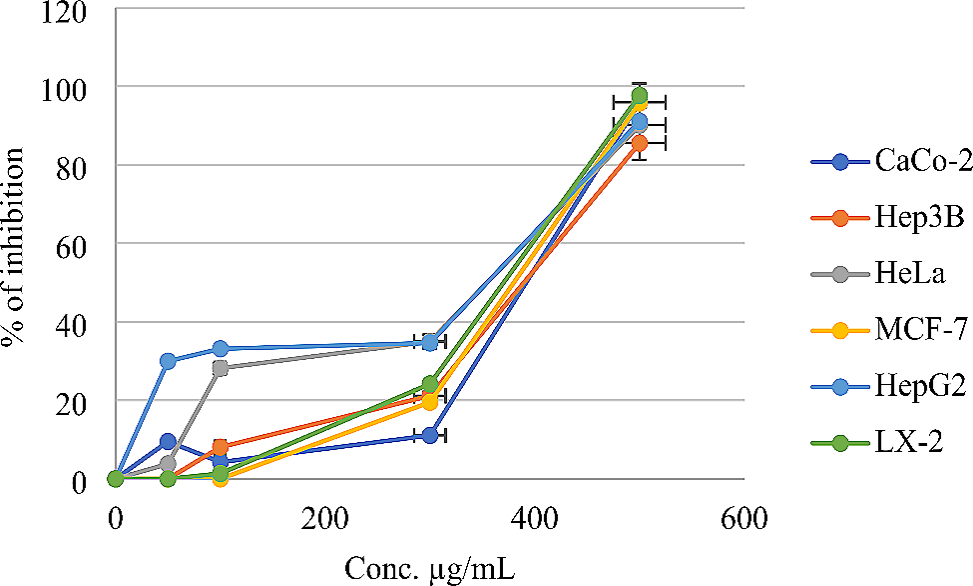
Cytotoxicity % of inhibition on Caco-2, Hep3B, HeLa, MCF-7, HepG2, and LX2 cells lines by *O. punonense* EO

In Fig. 2, the escalating concentrations of *O. punonense* EO demonstrated a proportional augmentation in the inhibition percentages of cell growth across the various cell lines. As the concentration of *O. punonense* EO increased, there was a discernible rise in the inhibitory effect on cellular proliferation. Notably, in Caco-2 cells, the inhibition percentage intensified with the ascending concentrations, reaching a zenith at the highest concentration assessed. Similarly, Hep3B cells exhibited a consistent escalation in growth inhibition as the concentration of *O. punonense* increased. This trend persisted across HeLa, MCF-7, HepG2, and LX-2 cell lines, signifying a dose-dependent response to the herb. These findings underscore the dose-sensitive nature of *O. punonense*, suggesting that higher concentrations of the plant are associated with a more pronounced inhibitory impact on cell growth across diverse cellular landscapes.

**Fig. 3:**
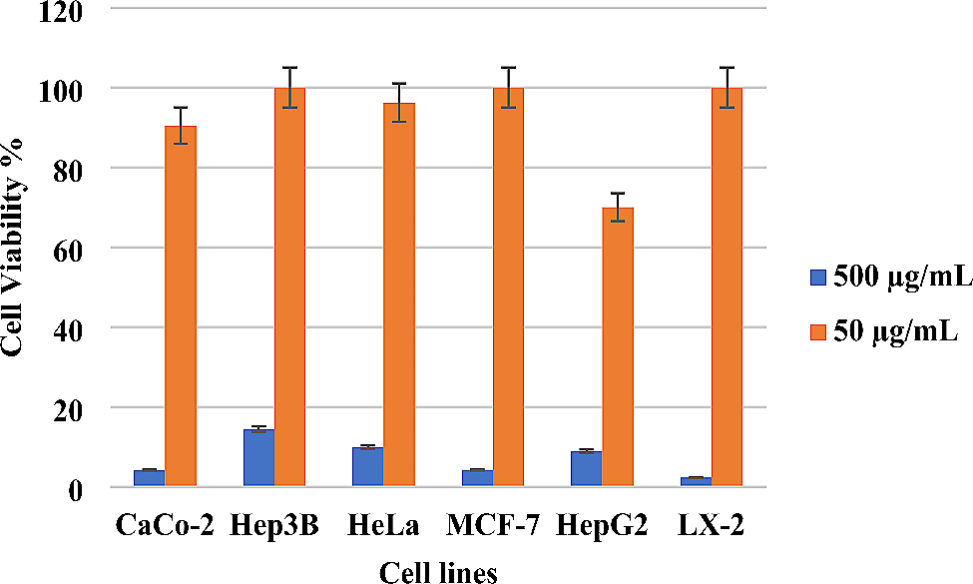
Caco-2, Hep3B, HeLa, MCF-7, HepG2, and LX2 cells viability (%) by *O. punonense* essential oil

In Fig. 3 our comprehensive assessment of cellular viability, *O. punonense* revealed intriguing concentration-dependent effects on the tested cell lines. At a concentration of 50 micrograms per milliliter, the herb exhibited a tolerable influence on cell viability, with no discernible toxic effects observed across the various cell lines under investigation. However, at a higher concentration of 500 micrograms per milliliter, a contrasting narrative unfolded. The viability of cells significantly decreased, signaling a pronounced toxic effect induced by the elevated concentration of *O. punonense*. This stark difference in cellular responses between the two concentrations underscores the critical importance of dosage considerations in harnessing the therapeutic potential of *O. punonense* EO with lower concentrations exhibiting a favorable impact on cell viability while higher concentrations necessitate cautious evaluation due to their evident cytotoxic effects. These findings highlight the nuanced balance required for leveraging the medicinal properties of *O. punonense* without compromising cellular well-being.

## Discussion

To the best of our knowledge, there is a noticeable lack of studies that were done to investigate the phytochemical characterization of *O. punonense*. Several studies were done to investigate the potential effects of the related species *O. syriacum* in Palestine [22, 23]. The current study identified the phytochemical compounds of the *O. punonense* EO to be principally carvacrol (57.4%), and to a lesser degree: p-cymene (6.66%), carvone (5.35%), α-pinene (4.9%), and d-terpinene (2.96%). In comparison with other *Origanum* species, Aligiannis and his colleagues demonstrated that carvacrol, terpinen-4-ol, linalool, sabinene, R-terpinene, and γ-terpinene were found as the major phytochemical compounds in *O. scabrum* and *O. microphyllum* [24].

The percentage yield of the EO of the same plant genus but for different species was 1.7% in Palestine for *O. syriacum* [22], and 0.6% in Jordan [25]. EO yield of *O. majorana* varied from 0.04 to 0.09% during the full-flowering stage in Tunisia [26]. *O. compactum* average yield was about 3.62% from the region of Meknes [27].

There is insufficient previous data regarding the antimicrobial effects of the *O. punonense* plant, so it was necessary to compare the current results with the studies on other *Origanum* species. The results showed that O. *punonense* had an impressive effect on *S. aureus, E. coli, K. pneumoniae, P. vulgaris, P. aeruginosa* (ATCC), and MRSA, compared to most other tested antibiotics. In fact, the demonstrated effects of *O. punonense* EO are better with variant folds than the used antibiotics except against *K. pneumoniae* and *S. aureus*, as Ciprofloxacin was better with MIC of 0.125 µL/mL and 0.78 mg/mL. Aligiannis and his colleagues [24] studied the effects of *O. scabrum*, which showed impressive impact on *S. aureus, S. epidermidis, P. aeruginosa*, *E. cloacae, K. pneumoniae, E. coli, C. albicans, C. tropicalis* and *T. glabrata* superior to several antibiotics. However, *O. microphyllum* had weaker effects on the same tested organisms, whereas it appeared to be completely inactive *against P. aeruginosa* and *K. pneumoniae*. It was suggested that carvacrol, a phenolic compound, possesses high levels of antimicrobial activity. While EOs from other *Origanum* species showed to possess high levels of antimicrobial activity, the EO inhibited the growth of all of the tested microbial strains [27].

Diabetic wound infections pose a significant challenge to healthcare providers worldwide. The *O. punonense* EO showed significant effects against all cultured bacteria compared to the used antibiotics. The EO of *O. punonense* was most potent against five strains of MRSA, with a lesser effect on *P. aeruginosa* obtained from diabetic foot patients, and more potent effects on Carbapenem-resistant *Enterobacterales*, *C. freundii* and *K. pneumoniae.* Berber and his colleagues showed that the EOs of *O. onites, O. onites oleum*, and *O. minutiflorum* at tested doses had considerably high antibacterial effects against *(A) pittii, (B) cereus, and K. pneumoniae* [28]. Moreover, the EOs of *O. onites, O. onites oleum*, and *O. minutiflorum*, had bactericidal activity against *(A) pittii, and K. pneumoniae* but bacteriostatic activity against *(B) cereus* [28].

It is worth mentioning that the phenolic carvacrol, the main component of *O. punonense*, was reported as a biocidal compound that resulted in bacterial membrane perturbations and leakage of intracellular ATP and potassium ions with ultimately cell death [29]. However, other minor components, such as γ-terpinene identified in the current study, displayed possible interaction between the substances that could ultimately affect the antimicrobial activities [30].

The EO of *Origanum* showed wound healing potential in rodents when blended with other biopolymers, including chitosan, alginate, gelatine, or collagen, to give active films or nanofibers with antioxidants and anti-inflammatory or antimicrobial activities. Rodent wounds showed better collagen deposition, enhanced fibroblast proliferation, and a faster closure rate [31].

Cancer is a leading cause of death and is responsible for one in eight deaths worldwide. There is rapidly accumulating scientific research encouraging the use of herbs as complementary medicine as an anticancer agent, especially for advanced cancer. So, it was important to investigate the antiproliferative effect of *O. punonense* EO. The results showed an impressive, nearly complete effect on all cell lines. This is in harmony with several previous studies that found evidence for the antiproliferative of plants related to the genus *Origanum* [32]. Other studies investigated the effect of *O. vulgare* EO, which was less effective in the MCF-7 cell type. The increase in the EO concentration did not enhance the cell growth inhibition. In the HT-29 cell line, the EO was significantly more effective (cell growth inhibition of 60.8%) and also presented the same characteristic of inducing cytotoxicity at 50 micrograms per milliliter and the non-enhancement of cell growth inhibition values with the increase of EO concentration. However, *O. punonense* EO showed gradual enhancement with increasing the concentration. These results suggest that the EO could have a selective activity, therefore offering an opportunity to investigate its use as anticancer agent. They attributed this to the action of its principal phenolic components, carvacrol, and thymol, which exhibit significant anticancer [33]. The same phenolic compounds were found in the current study as the major components of *O. punonense* EO. Arunasree demonstrated the anticancer effects of carvacrol in MDA-MB 231, a human metastatic breast cancer cell line. These authors showed that carvacrol-treated cells exhibited prominent morphological changes like cell shrinkage with rounding of cells and formation of membrane blebs characteristic of apoptosis [34]. Other studies have suggested that carvacrol might be potentially helpful in counteracting free radical-mediated injuries and in DNA damage by the ability to enhance the levels of antioxidants along with its anti-lipid peroxidative activity; what can be a beneficial action of carvacrol against pathological alterations like melanogenesis and cancer [34].

In brief, the broad spectrum of antimicrobial properties exhibited by *O. punonense* EO, particularly against microorganisms responsible for diabetic foot infections, along with its ability to inhibit cell growth, position it as a highly promising candidate for treating infections in immunocompromised patients, such as those with cancer and diabetes mellitus.

A limitation of this study is that it did not investigate the modes of action of antibacterial and antiproliferative activities of this plant. Also, these biological activities were not investigated in the specific phytochemical components of the plant. Moreover, we analyzed only the plant’s EO, so we did not compare these activities with other types of extracts. However, this was a leading study in which the biological activities of this plant were studied. Further investigations are warranted to decipher more about these findings.

## Conclusion

The current research is the first to explore the biological and phytochemical potentials of this plant species found in Palestine. The present results revealed the presence of many phytochemicals in the EO extracts of *O. punonense*. The *O. punonense* EO showed a very potent potential cytotoxic activity against LX-2 and Caco-2 cancer cells with inhibition activity of 97% and 96%, respectively. Moreover *O. punonense* EO showed a potent antimicrobial effect against *S. aureus*, *K. pneumoniae, P. vulgaris*, *P. aeruginosa*, *C. albicans* and impressive effect on Carbapenem-resistant *Enterobacterales*, *C. freundii*, *K. pneumoniae*, and MRSA that obtained from diabetic foot patients, and in most cases it was more effective than the positive controls: Ciprofloxacin, Ampicillin and fluconazole. These findings indicate that the *O. punonense* EO collected from Palestine is a promising natural source of potent biological activity as an antimicrobial and anti-tumorigenic agent. It can be used in future pharmaceutical formulations and as a treatment strategy for infection and cancer.

## Data Availability

The data used to support the findings of this study are included within the article.
